# The experience of Australian general practice patients at high risk of poor health outcomes with telehealth during the COVID-19 pandemic: a qualitative study

**DOI:** 10.1186/s12875-021-01408-w

**Published:** 2021-04-08

**Authors:** Sara Javanparast, Leigh Roeger, Yuen Kwok, Richard L Reed

**Affiliations:** Discipline of General Practice, Flinders University of South Australia, Adelaide, Australia

**Keywords:** Telehealth, General practice setting, Covid-19 pandemic, People at high risk of poor health outcomes

## Abstract

**Background:**

The emergence of the COVID-19 pandemic has raised concerns about the potential decrease in access and utilisation of general practice services and its impact on patient care. In March 2020, the Australian Government introduced telehealth services to ensure that people more vulnerable to COVID-19 do not delay routine care from their general practitioners. Evidence about patients’ experience of telehealth and its impact on patient care is scarce. This study aimed to investigate the experience with telehealth by Australian general practice patients at high risk of poor health outcomes during the COVID-19 pandemic.

**Methods:**

Semi-structured telephone interviews were conducted with 30 patients from nine general practices in metropolitan Adelaide (May–June 2020). Participants were identified by their regular doctor as being at high risk of poor health outcomes. Interviews sought participants’ perspectives and experiences about telehealth services in the general practice setting during COVID-19, and the value of offering continued telehealth services post pandemic. Interviews were recorded and transcribed verbatim. Data were analysed using a coding structure developed based on deductive codes derived from the research questions and any additional concepts that emerged inductively from interviews.

**Results:**

Participants expressed satisfaction with telehealth including convenient and timely access to general practice services. Yet, participants identified challenges including difficulties in expressing themselves and accessing physical exams. Prescription renewal, discussing test results and simple follow-ups were the most common reasons that telehealth was used. Telehealth was mainly via phone that better suited those with low digital literacy. Participants indicated that an existing doctor-patient relationship was important for telehealth services to be effective. Subjects believed that telehealth services should be continued but needed to be combined with opportunities for face-to-face consultations after the COVID-19 pandemic was over.

**Conclusions:**

The expansion of telehealth supported access to general practice including chronic disease management during the COVID-19 pandemic. In the future, telehealth in Australia is likely to have a stronger place in primary healthcare policy and practice and an increased acceptance amongst patients.

**Supplementary Information:**

The online version contains supplementary material available at 10.1186/s12875-021-01408-w.

## Background

The emergence of the COVID-19 pandemic has raised concerns about the potential decrease in access and utilisation of general practice services and its impact on patient care. Older people and those with chronic diseases are at higher risk of complications and death from COVID-19 [1, 2]. Patients in higher risk groups are encouraged to socially isolate and avoid public places but this may result in avoidance of routine general practice care for health problems.

Literature on access to health services during pandemics for vulnerable populations is sparse. Evidence from the 2002–2004 SARS outbreak suggests chronic-care hospitalisations for diabetes dropped during the crisis but significantly increased afterwards, a concern that is highly relevant to the COVID-19 pandemic [3]. Reasons for sub-optimal chronic disease management during emergencies include diversion of healthcare resources, interruption to routine care and medication supply, increased stress, and changes in activity levels [2].

During Covid-19, many countries implemented or expanded telehealth services to facilitate continued access to primary care and general practice services. The telehealth services provided during Covid-19 were to enable access to services especially general practice consultation from home via telephone or by using technology for video conferencing. A study by Hollander and Carr (2020) suggested urgent action to promote and expand telehealth to protect patients, clinicians and community from exposure [4]. The UK implemented programs to ‘support GPs in providing telephone-based or video-based consultations, and advice for outpatients’ during Covid-19 [5]. Likewise, the USA responded to the Covid-19 by the promotion and use of telehealth in primary health care setting (to provide telephone or video consultation, and remote monitoring of patients health) with reimbursement provided by Medicaid for some types of live videos [6]. In Australia, to ensure that people did not delay routine care from their general practitioners (GPs) during COVID-19, in March 2020 the Australian Government introduced temporary telehealth services (patient/doctor consultation via telephone or video) targeted to people more vulnerable to COVID-19 [7–9]. These services were offered at no charge to the patient (bulk billed). The key aim was to facilitate access to essential health services while reducing risk of exposure to COVID-19 [8].

A survey of over 1000 GPs conducted by the Royal Australian College of General Practitioners found that nearly all (99%) GPs were offering telehealth services during Covid-19, while also continuing to offer face-to-face consultations [10]. Medicare data shows that out of 35.2 million GP consultations (levels A to D) in Australia between April and June 2020, 34% were via telehealth [11]. The majority (97%) of telehealth consultations were by telephone with videoconferencing representing only a very small (3%) proportion of telehealth consultations [11]. Consumer survey also found positive views to telehealth with 43% of respondents preferring to have their usual appointments by telehealth during the COVID-19 outbreak [12].

The provision of telehealth on an ongoing basis is supported by professional bodies [13] and consumer organisations [12, 14]. In July 2020 the Australian government restricted access to Medicare-subsidised telehealth services to only patients who had a face-to-face consultation with their regular GP in the past 12 months, those living in COVID-19 hot spots, infants under 12 months and homeless people [15]. While some professional bodies welcomed this change as a way to prevent “low-value pop-up telehealth services” [16] there are concerns that tighter restrictions may exclude some high-risk patients.

In November 2020, the Australian Health Minister confirmed that telehealth would remain in place after the Covid-19 pandemic [17]. There has been minimal research on the impact of these changes on clinical care or patient satisfaction with the services provided. This study investigated the experience with telehealth of Australian general practice patients at high risk of poor health outcomes during the COVID-19 pandemic.

## Methods

We conducted semi-structured telephone interviews with 30 patients from nine general practices in metropolitan Adelaide (May–June 2020). The patients were former participants of a clinical trial of enhanced general practice services (Flinders QUEST) conducted in 2019. Flinders QUEST was designed as a clustered randomised control trial with 20 general practices in Adelaide, South Australia. The trial aimed to assess the effectiveness and cost-effectiveness of four enhanced services including patient enrolment to a preferred GP, longer appointment time, improved patient follow-up after hospitalisation and same day appointments for children and young people.

The eligibility criteria for Flinders QUEST were that patients had at least three on-site GP visits at a GP practice within the previous two years and were identified by their GP to be at high risk of poor health outcomes. Participants were drawn from two cohorts: adults 18–64 years of age with two or more chronic diseases and people 65 years of age and above.

The Flinders QUEST trial evaluation involved interviews with 45 patients attending 10 general practices from the intervention group. Of 45 patients participating the original trial evaluation, for this study we approached 33 potential participants who had agreed to further contact with the researchers. An invitation letter was mailed to these 33 patients followed by phone calls 1 week after the mail-out. Of the 33 people approached, 30 agreed to participate (average of 3–4 patients per practice) and a telephone interview was arranged. Participants provided verbal consent for participation and for recording of interviews. The previous relationships with participants through the Flinders QUEST facilitated recruitment and rapport building.

An interview guide was developed, discussed and refined by the research team. The guide included questions on participants’ perspectives and experiences with telehealth services in the general practice setting during Covid-19, the benefits and challenges related to telehealth, and participant’s views about the continuation of telehealth services post pandemic. Interviews took approximately 20–30 min.

Interviews were recorded and transcribed verbatim and imported into NVivo-12 software for data management and coding. A coding structure was developed based on deductive codes from the research questions. We also generated codes inductively to capture additional concepts emerged from interviews. The coding structure were reviewed and refined to group codes that were related to similar themes and a thematic data analysis approach was used.

## Results

Participants were between 54 and 88 years of age (17 females and 13 males) and had 2 or more chronic condition for example diabetes, cancer, musculoskeletal issues and mental health. Key study findings are presented below:

### Access to general practice services and management of health conditions

Participants on average reported 2–3 GP consultations (ranged from 0 to 6) either face-to-face or by telephone since COVID-19 commenced (an approximate 3-month period). A small number of participants reported that they decided to postpone or stop making new GP appointments for non-urgent medical issues.

Consultations by telephone were viewed as an enabling factor in accessing general practice care:

*I’ve found the medical fraternity has really stepped up to the plate, as far as making available these [phone] appointments, and to keep going the regular health checks that they have with people. I’m pretty impressed… (female, 68 yrs. old)*

*Well, it hasn’t been the same obviously, but I haven’t felt as though I’ve been deprived… certainly on the doctor’s part they were available, I’ve always been accommodated. (male, 73 yrs. old)*

Out of 30 participants, only two reported longer waiting time and less frequent GP consultations:

*I’ve got to physically go there, they would only allow so many people into surgery, having to have the constant temperature [checks]. It’s all valid but all these extra things…that didn’t enable us to see him [GP] as frequently as we normally would have been before COVID. (male, 59 yrs. old)*

Twenty-nine out of 30 participants felt that, although their health had been managed differently during COVID-19 it had been managed quite well:

*I think my health has been managed very well. If I needed, I know that the doctors would be available at the end of the phone, just the way things have gone along, I haven’t had any big problems. (female, 80 yrs. old)*

Three participants even noted an improvement in their health management:

*I actually think it’s better… people are much more aware of health, cleanliness. We’ve had less cases of flu. People are more thinking about how we react with each other, the distance, it’s made us think about a lot more things and what it impacts on. (female, 68 yrs. old)*

### Experience of telehealth services

Twenty-five participants utilised telehealth (at least once) for GP consultations. Of the five who did not use telehealth, three did not need a GP appointment and two did need but preferred face-to-face GP consultations due to their health conditions.

Those who had a telehealth consultation were very positive about it:

*If we needed something we rang there and he [GP] rang us on the phone. If it was something that he wasn’t sure about or needed to check out – an appointment given to us and we had to go in and come straight out. It was quite good, a new standard. (female, 77 yrs. old)*

The availability of telehealth was viewed as potentially increasing the frequency of GP visits ‘*In fact, I’m probably seeing him [GP] more now *via* the phone’ (female, 56 yrs. old),* or saving travel time *‘It would save the person having to go into the surgery’ (female, 80 yrs. old).*

Most felt that phone consultations were not rushed ‘*business was done, what’s the point in hanging around. I didn’t need any more time than what I had from the doctor’ (female, 68 yrs. old).*

Prescription renewal, discussing test results and simple follow-ups were the most common reasons for which telehealth was used:

*It helps if we don’t necessarily have to go face-to-face all the time, but a phone call. A renewal of prescriptions you’ve been on for a long time, and you’re finding them working well. (female, 68 yrs. old)*

*It was just a 3-monthly diabetes check-up so it was nothing that couldn’t have been done over the phone anyway. (male, 64 yrs. old)*

The availability of telehealth was highly appreciated by one participant who was diagnosed with cancer and required cancer investigations and treatments:

*I was extremely grateful for those phone calls. It is, of course, not as good as a face-to-face because they can't see how you're looking and how you're actually doing. Nevertheless, I would have been lost without the phone calls. I appreciated that hugely. (female, 77 yrs. old)*

There were, however, differing views on the usefulness of telehealth for mental health issues. While one participant felt: *‘if you had mental health problems that would probably be a difficult one to talk about over the phone, you might need to speak to somebody.’ (female, 68 yrs. old),* another participant stated: *‘I see the benefit in the mental health space for argument’s sake where people are able to talk…to just be there privately in your own room with your computer talking to the person, it does assist a lot’ (male, 59 yrs. old).*

A few participants who normally paid an additional charge (gap fee) for face-to-face GP visits appreciated the no-additional fee services (bulkbilling) mandated by Medicare for telehealth services:

*The GP appointments have been really good because they’re bulkbilling, also I’ve been able to get in easily for the phone appointments. Our doctor’s surgery, they’re charging us, and now we’re not being charged, that’s a relief. (female, 56 yrs. old)*

Many participants believed that ‘*familiarity of the doctor with patient’* and their previous relationships with the GP (for some this was over 20 years) was crucial for telehealth communication:

*Because my doctor knows me, I could say, “I need antibiotics or prescription”, and he says, “Yeah, okay”… but if you are talking by phone with a doctor that hasn’t seen you or doesn’t know you. That’s a big difference, isn’t it? (female, 68 yrs. old)*

*I’ve been going to my GP for a long time so he really knows me and he knows when I say I need stronger pain relief, he knows I’m in a lot of pain. That’s why I feel we’re fine on the phone. (female, 70 yrs. Old)*

Finally, despite an emphasis in policy documents on videoconferencing as the preferred mode of communication, participants in this study were only offered telephone consultations with their GPs. For older participants with lower digital literacy telephone conversation was more convenient’ *I don’t get on with the computer, I’m a bit old-fashioned’ (female, 69 yrs. old)*, however a few mentioned that they would have preferred videoconferencing but this wasn’t offered:

*If it was perhaps online, such as Microsoft Meeting or Zoom, I probably would appreciate that a bit better, some GPs are offering just a phone consultation, I don't think that's satisfactory. (female, 76 yrs. old)*

### Opportunity for face-to-face consultations

Participants appreciated that they were still able to have face-to-face GP appointments if needed. Of 30 participants, 26 had had a face-to-face consultation. The COVID-19 safety measures adopted by general practices, mitigated patient’s fear of contracting the virus and this was not viewed as a major barrier to attending in person to the practice.

*I felt quite comfortable, they were not taking a lot of people in, it was very staggered…you weren’t sitting with a lot of people in a waiting room with everyone coughing and sneezing over you. The practice did it very well, I had no concern at all. (female, 80 yrs. old)*

*I preferred to go there, I found that when you see the doctor you can sit there, you can read his face and I guess he can read yours. I found by phone it was less personal, less invasive, less whatever. (male, 73 yrs. old)*

### Continuation of telehealth services

Participants felt that telehealth should be continued post COVID-19 for issues that do not need physical contact:

*Absolutely, if I’m just doing that 6 or 12-monthly blood test thing, I’d be more than happy for him to mail me the paperwork and ring up for a report - no dramas. It’s only when I have something that I feel I need to see him about. (male, 73 yrs. old)*

*Unless it’s urgent I reckon the phone would be just fine after this pandemic period, especially for something like prescriptions or simple things. (female, 54 yrs. Old)*

## Discussion

Telehealth has been advocated in Australia for many years to support access to health services especially in rural and remote areas [18]. The COVID-19 pandemic required a rapid adoption of telehealth to reduce the likelihood of virus transmission in patients and health providers, and to ensure that access to general practice services was maintained. Our study found that the telehealth model appears to have mitigated many of the adverse consequences of Covid-19, providing access to routine services. These findings are supported by other studies showing patient and caregivers satisfaction and the role of telehealth in facilitating continued access to care [19, 20].

The convenience and timely access to general practice care was particularly appreciated for services that do not usually require physical contact including repeat prescriptions, reporting of test results and monitoring of less complex health conditions. Continued access to face-to-face consultations (along with telehealth) was felt by patients to be the optimal approach. However, a few patients chose not to use telehealth instead preferring face-to-face consultations. Consistent with other studies [5, 21], our participants identified some challenges in using telehealth including difficulties in expressing themselves and undertaking physical examination by GPs when needed.

Our findings support the importance of provider-patient relationships for telehealth to be effective. New changes imposed by the government in July 2020 restricting access to telehealth to those who have an established relationship with a GP has already raised debates about its impact on equity of access to general practice services. Whether having a ‘face-to-face consultation with the regular GP in the past 12 months’ as identified in recently policy change [16] is a true measure of ‘doctor-patient relationship’ and the impact of this policy on the equity of access and patients satisfaction requires further investigation but appeared to work well with this group of older patients with chronic health conditions who already had established relationships with GPs.

The use of telephone rather than video consultation (which is the preferred mode of consultation of the Australian Government) seems to be more acceptable for patients in this study. It may be partly due to lower level of digital literacy amongst older patients. If telehealth technologies are to be included in the future health communication strategies, further investment and training to facilitate both health providers and patients in using such technologies will be important [5].

A combination of telehealth and face-to-face services that allows choice of mode to obtain general practice services would facilitate continuity, accessibility and equity of care. The flexibility in modes of service delivery is particularly important for some older patients with chronic health conditions whose conditions are impacted by broader social determinants of health. As noted by Fisk et al. (2020), ‘telehealth must not be seen as an alternative form of healthcare.’ Lessons learnt during the current pandemic should be incorporated into future routine practices as well as professional training curricula [5, 22].

The study participant sample represented a cohort of patients likely to be at greatest risk of poor health outcomes from COVID-19 and from disruptions to their regular healthcare caused by COVID-19. The benefits of telehealth reported by our participants however may be not be generalisable to people without an established relationship to a general practitioner. In addition, the selection criteria excluded people who were not fluent in English. The communication barriers experienced by this group may make it more difficult for them to utilise telehealth services and needs further study.

Overall, this blended model appeared to be satisfactory to this population group avoiding some face-to-face consultations and enhancing access for this vulnerable population. This study supports the use of this model beyond the Covid-19 pandemic.

## Conclusions

The expansion of telehealth supported access to general practice including chronic disease management during the COVID-19 pandemic amongst our study participants. This suggests the potential for telehealth to play a stronger role in general practice services in the future and therefore a need for further studies on its use on broader patient groups and consultation needs.

## Supplementary Information

**Additional file 1.**

## Data Availability

The data used and analysed during the current study are available from the corresponding author on reasonable request.
