# Supplementary material for: The experience of Australian general practice patients at high risk of poor health outcomes with telehealth during the COVID-19 pandemic: a qualitative study
Source: BMC Fam Pract. 2021 Apr 8;22:69. doi: 10.1186/s12875-021-01408-w (PMC8031338; doi:10.1186/s12875-021-01408-w)
Supplement: Supplementary file 1 — Additional file 1. [file 12875_2021_1408_MOESM1_ESM.docx]

**Access to and utilisation of health services by older people and people with chronic health conditions during the COVID-19 pandemic**

**Interview Schedule**

**(Via phone or video-call)**

- Thank the participant for taking part in the interview
- Explain the interview aim and process
- Ask if s/he has any question
- Ask for verbal consent

The interview will be started with EQ-5D questions. This will be followed by open-ended questions:

1. Can you tell me a bit about changes that may have happened to your daily routines because of COVID-19 situation over the last couple of months?

[Probes: your diet and sleep pattern, exercise, communication and psycho-social support from family, communities or services]

- 1. How do you feel these changes affected your health?

1. Have you visited your GP during this time? (can you recall when was the last time you saw your GP?)
   1. If yes, was it part of your regular consultations? Do you visit your GP with the same frequency as before?
   2. Have you had to cancel any of your appointments? Why?

[the practice cancelled the appointment or you made that decision]

[probes: fear of contracting COVID-19, balancing the risk of COVID-19 versus risk of maintaining health and managing the chronic condition)

- 1. When are you planning to see your GP next time?

1. How do you feel about the support you received from your general practice (nurse, GP or other staff) since COVID-19 situation started?
   1. Have you had any contact from the practice?
   2. Have you been offered telehealth consultations?
   3. What have been your experience of telehealth (if any)? Better or worse than face-to-face visits?
   4. In the future (i.e. after COVID-19 pandemic), would you feel more convenient to use telehealth service or prefer to go back to face-to-face consultations with your GP?

[Can you think of any values that telehealth would add to regular face-to-face consultations? E.g. following up test results or requests for prescriptions]

- 1. Has there been any other outreach services that you have used or are aware of?

1. How about specialist care? Have you had any appointment with your specialist during the COVID-19 pandemic?
   1. If yes, did it go ahead or cancelled? Why?
   2. Do you usually visit your specialist at a private office or at hospital? Has it had any impact on your decision to go to or cancel your appointment?
   3. Have you had any experience of telehealth with your specialist/s? if yes, can you share your experience with us?
2. You may need frequent prescriptions for the medications you use for your health condition. Have you experienced any problem in that regard? Have you received any support? (e.g. getting prescription from your GP or specialist, going to pharmacy)
   1. How about blood test, etc? do you need to do regular tests? Have you been able to keep your routines during this time?
3. Did you have to go to the emergency department or admit to hospital over the last couple of months?
   1. If yes, can you explain what was it for and what have been your experience? Have you noticed any changes from before the COVID-19 situation?
4. Overall, how do you think your health has been managed during the COVID-19 situation? The same, better or worse? Why?
5. Is there anything you think can be done differently to make it easier for you to get access to the health services you need during COVID-19 situation?
